# Clinical implementation of an algorithm for predicting exacerbations in patients with COPD in telemonitoring: a study protocol for a single-blinded randomized controlled trial

**DOI:** 10.1186/s13063-022-06292-y

**Published:** 2022-04-26

**Authors:** Pernille Heyckendorff Secher, Stine Hangaard, Thomas Kronborg, Lisa Korsbakke Emtekær Hæsum, Flemming Witt Udsen, Ole Hejlesen, Clara Bender

**Affiliations:** 1 Department of Health Science and Technology, Aalborg University, Fredrik Bajers Vej 7C, 9220 Aalborg East, Denmark; 2 Department of Nursing, University College of Northern Denmark, Selma Lagerløfs Vej 2, 9220 Aalborg East, Denmark

**Keywords:** Chronic obstructive pulmonary disease, Disease exacerbation, Physiological monitoring, Randomized controlled trial, Clinical decision support systems, Telemedicine, Forecasting, Machine learning, Health literacy

## Abstract

**Background:**

Acute exacerbations have a significant impact on patients with COPD by accelerating the decline in lung function leading to decreased health-related quality of life and survival time. In telehealth, health care professionals exercise clinical judgment over a physical distance. Telehealth has been implemented as a way to monitor patients more closely in daily life with an intention to intervene earlier when physical measurements indicate that health deteriorates. Several studies call for research investigating the ability of telehealth to automatically flag risk of exacerbations by applying the physical measurements that are collected as part of the monitoring routines to support health care professionals. However, more research is needed to further develop, test, and validate prediction algorithms to ensure that these algorithms improve outcomes before they are widely implemented in practice.

**Method:**

This trial tests a COPD prediction algorithm that is integrated into an existing telehealth system, which has been developed from the previous Danish large-scale trial, TeleCare North (NCT: 01984840). The COPD prediction algorithm aims to support clinical decisions by predicting the risk of exacerbations for patients with COPD based on selected physiological parameters. A prospective, parallel two-armed randomized controlled trial with approximately 200 participants with COPD will be conducted. The participants live in Aalborg municipality, which is located in the North Denmark Region. All participants are familiar with the telehealth system in advance. In addition to the participants’ usual weekly monitored measurements, they are asked to measure their oxygen saturation two more times a week during the trial period. The primary outcome is the number of exacerbations defined as an acute hospitalization from baseline to follow-up. Secondary outcomes include changes in health-related quality of life measured by both the 12-Item Short Form Survey version 2 and EuroQol-5 Dimension Questionnaire as well as the incremental cost-effectiveness ratio.

**Discussion:**

This trial seeks to explore whether the COPD prediction algorithm has the potential to support early detection of exacerbations in a telehealth setting. The COPD prediction algorithm may initiate timely treatment, which may decrease the number of hospitalizations.

**Trial registration:**

NCT05218525 (pending at clinicaltrials.gov) (date, month, year)

## Introduction

### Background

Chronic obstructive pulmonary disease (COPD) imposes a substantial economic and social burden globally [1, 2]. The global prevalence was estimated to 251 million cases in 2016 [3]. COPD is the third leading cause of morbidity and mortality worldwide [4] and is responsible for 3 million deaths annually [5]. The disease is projected to increase further over the coming decades with an estimated mortality of 4.5 million deaths annually by 2030 [6] and 5.4 million deaths annually by 2060 [2].

According to Global Initiative for Chronic Obstructive Lung Disease (GOLD) COPD refers to a group of progressive lung diseases characterized by not fully reversible airflow limitation that causes long-term breathing problems [2, 7]. In mild stages of COPD, the symptoms are present only by infections or by strenuous physical exertion. In the more severe stages of COPD, the symptoms are more prominent and will usually increase over time [8, 9]. Most patients with COPD experience periods where their symptoms vary from day to day [2]. However, sometimes patients also experience acute worsening of their usual symptoms, which are referred to as exacerbations. According to GOLD, an exacerbation is defined as “an acute worsening of respiratory symptoms that results in additional therapy” [7]. The frequency of exacerbations increases with the severity of COPD [10] and exacerbations contribute to the worsening of COPD at a long-term level [8, 11, 12]. Moreover, exacerbations are associated with increased mortality as well as impaired prognosis, decline in lung function, worsen cardiovascular comorbidities, and decreased health-related quality of life [8, 12, 13]. More than four out of five exacerbations can be managed outside the hospital with proper medication, whereas more severe exacerbations require ventilator support and hospitalization [7]. Early treatment is associated with faster recovery and decreased risk of emergency hospitalization [14]. However, patients with COPD often delay or fail to seek treatment during exacerbation [15], and it has been estimated that 40% to 70% of exacerbations remain unreported to healthcare personnel [14–16]. This highlights the importance of detecting exacerbations earlier in order to facilitate early and preventive treatment [14, 17].

### Rationale

Management strategies to prevent exacerbations include various pharmacological and non-pharmacological interventions [14, 18, 19]. However, the effect of these interventions remains moderate indicating the need for better management strategies [14, 18–21] that allow for earlier intervention while using scarce health care resources efficiently. Although telehealth interventions internationally show promising results related to admission rates and quality of life [15, 22–26], improvements in evidence are needed to come closer to identifying individual patients in risk of exacerbations in order to improve the outcomes of telehealth interventions [20, 21, 27]. In the TeleCare North trial, there were patients who benefitted greatly from having the telehealth system while others did not. Averaging this variability lead to the conclusion that patients experienced a non-statistically insignificant gain in health-related quality of life [28], increased sense of control, freedom, security, awareness of COPD symptoms [28–34] and that cost-effectiveness was most likely in the subgroup of patients with severe COPD [30]. In order to implement telehealth more effectively than by applying averaged results across clinical subgroups, machine-learning algorithms have been suggested as a way forward [35], e.g., by seeking to support clinical decisions in predicting exacerbations using patient physical measurements and other characteristics [27, 36–38].

Aalborg University has developed and optimized several COPD prediction algorithms for early detection of acute exacerbations that require hospitalization. These COPD prediction algorithms are based on both data from TeleCare North and a previous study conducted at the Department of Pulmonary Medicine at Aalborg University Hospital, Denmark [37–40]. The algorithms were based on mathematical features extracted from patients’ home measurements of pulse, blood pressure, and oxygen saturation. Since symptoms of an exacerbation are typically only present a few days prior to an exacerbation [10, 41] these algorithms underlined the importance of high-frequency measurements, to achieve the highest prediction accuracy [38, 41]. Experiences indicated that a frequency of at least 3 measurements each week, simple algorithms could achieve a sensitivity of 0.65 at a specificity of 0.95, whereas more advanced algorithms achieved a sensitivity up to 0.94 at a specificity of 0.95 [40]. It is therefore hypothesized that a prediction algorithm can reduce hospitalizations through facilitating an early and preventive treatment, which also can improve patients’ quality of life and lead to a greater economic effect of the telehealth intervention.

### Objective

In 2020, the Danish Government, Local Government Denmark, and Danish Regions initiated 15 signature projects to investigate, develop and test digital welfare solutions in the field of artificial intelligence [42]. This trial is established as one of these signature projects carried out on the initiative of Aalborg University, Aalborg Municipality, and the North Denmark Region. In this trial, the developed COPD algorithm from Aalborg University will be tested in an already implemented telehealth system. This algorithm is based on weekly measurements of blood pressure, pulse, and oxygen saturation from where the mean and variance are extracted. The COPD prediction algorithm aims to support clinical decisions by predicting exacerbations in patients with COPD based on physiological parameters. The trial is based on the hypothesis that by predicting exacerbations, the algorithm can reduce the number of acute hospitalizations and improve the health-related quality of life of patients with COPD and the cost-effectiveness of the telehealth solution. Hence, the primary objective of the study is to explore if the inclusion of an algorithm in a telehealth system can predict exacerbations and consequently reduce acute hospitalizations. The second objectives are to explore if inclusion of a prediction algorithm in a telehealth system leads to (1) improved health-related quality of life of patients with COPD and (2) improved cost-effectiveness of the telehealth solution.

## Methods

### Study setting

**Fig. 1:**
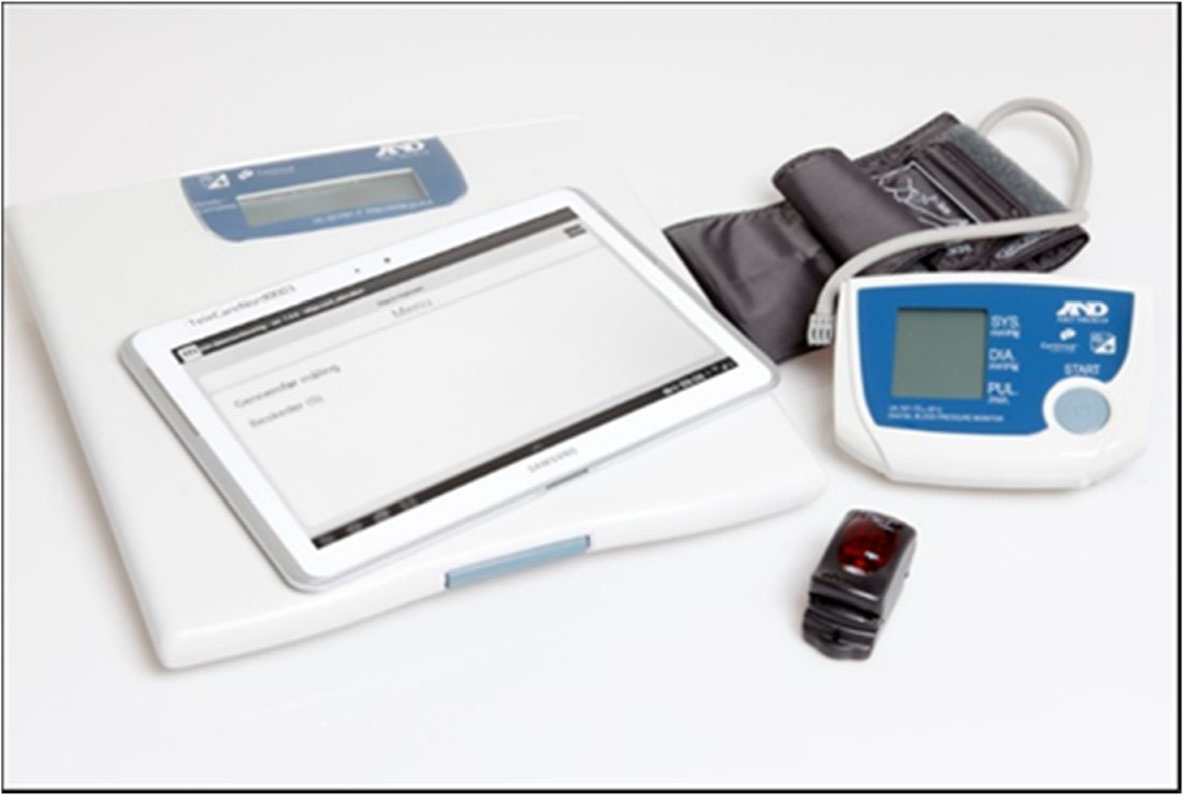
The telehealth system, Telekit, consists of a tablet, a fingertip pulse oximeter, a blood pressure monitor, and a scale

The TeleCare North trial was conducted between 2014 and 2015 [34] and its solution is currently operational in routine practice in the North Denmark Region. Patients with COPD are provided with an internet-based monitoring and treatment system, named Telekit [34]. The Telekit consists of a tablet (Galaxy TAB 2, 10.1, Samsung Electronics, Seoul, South Korea), a fingertip pulse oximeter (Nonin, Onyx II% SpO2, A and D Medical, Tokyo, Japan), a blood pressure monitor (Model UA-767, plus BT-C, Nonin Medical, MN, USA), and a scale (Precision Health Scale, UC-321PBT-C, A & D Medical, Tokyo, Japan) [43] (Fig. 1).

The commercial and CE-marked telehealth system represents an open-source platform that collects health data and provides remote care for the patients with COPD. The telehealth system is based on a mobile- and web-based healthcare platform of which, the mobile platform is used by patients with COPD to measure their vital signs (blood pressure, oxygen saturation, pulse, and weight) and answer questions related to subjective parameters (such as shortness of breath, color of mucus) at home twice a week. The measurements and questions are then transferred wirelessly to specialized COPD community nurses. The specialized COPD community nurses use the web-based portal to monitor the patients’ health data on a weekly basis (measurements are typically reviewed Monday or Thursday) based on preset fixed alarms [43]. These preset fixed alarms are triggered by “too high” or “too low” single values of physiological parameters (oxygen saturation, pulse, blood pressure, and weight) that can be changed according to the individual needs. The specialized COPD community nurses are responsible for monitoring the patients’ vital signs and provide telephone advices over distance, if the vital signs deviate from the patients’ normal expected values. Such advice may lead the patient to adjust medication or to see a doctor.

### Study design

This trial is a prospective, parallel two-armed randomized (1:1) controlled trial with a nested full economic evaluation with 6 months follow-up. The trial is expected to run from October 2021 to May 2022. Prior to randomization, participants must give written consent in accordance with the Helsinki declaration [44]. Data will be anonymized and stored in accordance with the Danish Data Protection Rules [45]. The trial has been approved by the Regional Ethical Committee for Medical Research in the North Denmark Region (no.: N-20200076) and the Danish Data Protection Agency. Any protocol modifications will only be implemented after approval from the Regional Ethical Committee.

The trial is single-blinded, as the participants are unaware of their group allocation. The specialized COPD community nurses will not be blinded, as they will know whether the COPD prediction algorithm supports them or not. Outcome assessment will not be blinded.

### Eligibility criteria

The trial population consists of patients with COPD who already use the telehealth system.

#### Inclusion criteria

Both men and women ≥18 years are qualified for inclusion. Furthermore, the participants must be diagnosed with COPD at any stage (no restrictions regarding the diagnostic period). The participants must also have a fixed residence in Aalborg Municipality.

#### Exclusion criteria

Participants are excluded if they are unable to monitor their vital signs or unable to complete study questionnaires.

### Recruitment

**Fig. 2:**
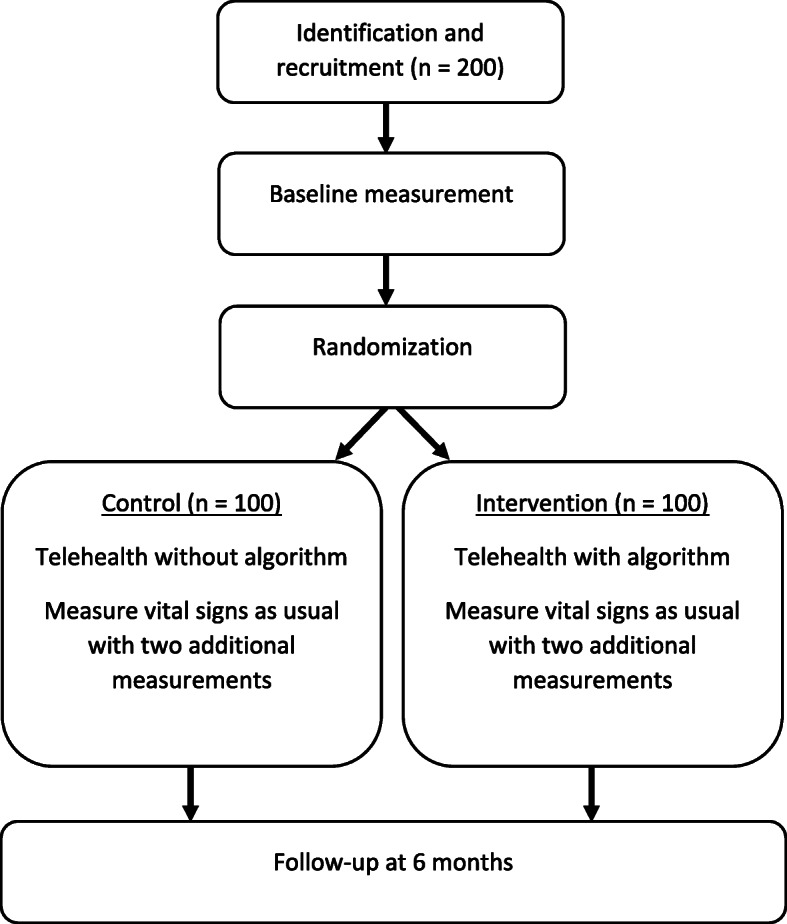
The trial procedure from identification and recruitment to outcome assessments at 6 months

The recruitment takes place in Aalborg Municipality. Specialized COPD community nurses identify eligible participants prior to randomization (Fig. 2). Subsequently, researchers will contact the participants to give them further information about the trial, answer potential questions, and guide them regarding the written consent form. A written consent form and questionnaires will be sent to eligible participants after receiving oral consent. If the participants still wish to participate in the trial, they will return the questionnaires and the written consent form via mail using a second pre-stamped envelope. The researchers’ telephone numbers are stated on the questionnaires giving the participants the opportunity to receive help in answering the questionnaires.

### Randomization

The participants will, by a computer-generated list be allocated randomly (1:1) across two treatment arms: an intervention group (telehealth with algorithm) or to a control group (telehealth without the algorithm). The allocation sequence will be concealed until the participants are enrolled and assigned to the two groups. The randomization will not be performed until the included participants have signed informed consent and completed baseline questionnaires. Researchers at Aalborg University will generate the allocation sequence, enroll the participants, and assign the participants to their respective groups.

## Intervention

All participants in both groups are encouraged to measure and transfer oxygen saturation, blood pressure, and pulse data three times per week. However, patients are also allowed to only measure and transmit oxygen and pulse data using the pulse oximeter if measuring blood pressure causes discomfort. Generally, the participants must measure their vital signs on fixed days in the week, starting either Monday or Thursday. Two monitoring schedules will occur depending on the participants measuring day. If the participants usually measure on Monday, they must perform additional measurements Wednesday and Friday. Whereas if the participants usually measure on Thursday, they must measure the additional measurements on Saturday and Monday. Thus, all specialized COPD community nurses will receive more data they can use in assessing the participants’ conditions during the trial period. The only difference between the two groups is that the specialized COPD community nurses monitoring the intervention group are aided by the COPD prediction algorithm, and the specialized COPD community nurses monitoring the control group are monitoring solely based on their professional opinion and the alarms implemented in the current telehealth system. The participants will be unaware of their group allocation. The specialized COPD community nurses will not be blinded. However, they are instructed not to tell the participants about their group allocation.

### Intervention group

Participants in the intervention group will receive the general offer of the telehealth intervention including the telehealth system with the implemented COPD prediction algorithm. The COPD prediction algorithm uses weekly measurements of pulse, oxygen saturation, and blood pressure. The algorithm alerts the specialized COPD community nurses based on the probability of exacerbation within the next three days, which is estimated by logistic regression [40]. The alarm will be implemented in the telehealth platform that the nurses are already used to and will be positioned next to already existing alarms (e.g. “low values of blood pressure” or “high values of pulse”). Prior to the trial, the specialized COPD community nurses will receive training in the extended monitoring and to the addition of the new alarm. All alarms, including the COPD prediction algorithm alarm, are decision support for the specialized COPD community nurses while inspecting data and assessing the participants’ situation. The specialized COPD community nurses solely decide whether to contact the participants and/or their general practitioner.

Depending on how often the patients measure the various physiological parameters, the algorithm will predict risk of exacerbation based on one of the following two scenarios:

*Scenario 1:* The COPD prediction algorithm uses all three physiological parameters, if the patient has three weekly measurements of both pulse, oxygen saturation, and blood pressure. In this case, the COPD prediction alarm is based on the following:

- Pulse: The average of the measurements from the last week
- Oxygen saturation: The variance of the measurements from the last 2 weeks
- Diastolic blood pressure: The variance of the measurements from the last week

*Scenario 2*: The COPD prediction algorithm uses only two physiological parameters, if the patient only has three weekly measurements of pulse and oxygen saturation. This is due to insufficient predictive information at less than three weekly blood pressure measurements. In this case, the COPD prediction alarm is based on the following:

- Pulse: The average of the measurements from the last week
- Oxygen saturation: The variance of the measurements from the last 2 weeks

### Control group

Participants in the control group will continue with the general offer of the telehealth intervention without the COPD prediction algorithm. These participants are instructed to perform the same frequency of measurements as the intervention group.

### Measures to ensure minimal loss to follow up

A few measures will be taken to ensure minimal loss to follow-up. First, the specialized COPD community nurses are in contact will the participants continuously throughout the trial as part of the telehealth intervention. If the specialized COPD community nurses observe that a participant misses several measurements, he/she will contact the participant per telephone and encourage the participant to keep performing the additional measurements. If a participant is unable to return the questionnaires by mail, a researcher will pick up the letter at the participant’s home address to ensure complete follow-up.

### Criteria for discontinuation

No risks are expected to be linked to participation in the trial. On the contrary, it is expected that the quality of the telemonitoring will be increased due to the increased attention and data. However, should any adverse events occur, they will be recorded in the trial master file in REDCap with information on whether the primary investigator attributes association with the trial. Participants can always withdraw consent and will then be excluded from the trial.

### Outcomes

This study has one primary outcome, two secondary outcomes, and five explorative outcomes.

#### Primary outcome

- The primary outcome is the between-group difference in the number of observed exacerbations defined as an all-cause acute hospitalization from baseline to follow-up. The trial hypothesizes that integrating a COPD prediction algorithm into the telehealth system will lead to a significantly lower number of exacerbations through early identification and timely preventive treatment

#### Secondary outcomes

The secondary outcomes include:

- The change in health-related quality of life (HRQoL) using the 12-item Short Form Health Survey (version 2) (SF-12v2) [46] and the EuroQol-5 Dimension Questionnaire (EQ-5D-5L) [47] at the individual level from baseline to follow-up at 6 months. The trial hypothesizes that the difference in HRQoL from baseline to follow-up in both groups decrease since the participants have lived 6 months longer with COPD. However, it is expected that the decrease in HRQoL will be lower for the intervention group compared to the control group. Both questionnaires have previously been validated in the Danish population [48–50].
- The incremental cost-effectiveness ratio or ICER measured as the total cost per quality-adjusted life years (QALY) gained for the cost categories included in the study from baseline to follow-up at 6 months. It is hypothesized that the cost of hospital contacts will decrease, but it is unknown whether this cost is offset by an increase in other cost categories such as community care

#### Explorative outcomes

- To assess the participants’ health literacy level at baseline using the European Health Literacy Survey Questionnaire (HLS-EU-Q16) [51], supported by further assessment with the Danish Test of Functional Health Literacy in Adults (the Danish TOFHLA) [52] during the trial period to examine whether the effect of the COPD prediction algorithm is similar in patients with COPD, regardless of health literacy level
- To examine whether the specialized COPD community nurse’s estimate of the individual participant’s level of health literacy influences the effect of the COPD prediction algorithm
- To evaluate the specialized COPD community nurses’ experiences with the usability of the COPD prediction algorithm using interviews
- To evaluate the participants’ experiences with the usability of the telehealth system after trial completion using the Danish Telehealth Usability Questionnaire (D-TUQ) questionnaire [53]
- To evaluate the participants’ experiences with data ethical aspects after trial completion using qualitative research interviews

The SF-12v2, the EQ-5D-5L, HLS-EU-Q16, and questions about demographic characteristics are sent to the participants by letter at baseline. The D-TUQ, SF-12v2, and EQ-5D-5L are sent at follow-up (Table 1). If a participant is unable to return the questionnaires by mail, a research assistant will pick up the letter at the participant’s address to promote complete follow-up.

**Table 1:** An overview of the questionnaires that the participants receive at baseline, intermediate, and after 6 months follow-up

Name of the questionnaire	Time point for each outcome
The 12-item Short Form Health Survey (version 2)	Baseline, 6 months follow-up
The EuroQol 5-Dimension questionnaire	Baseline, 6 months follow-up
The European Health Literacy Survey Questionnaire	Baseline
Questions about demographic characteristics	Baseline
The Danish Test of Functional Health Literacy in Adults The	Intermediate
Danish Telehealth Usability Questionnaire	6 months follow-up

During the trial period, the level of health literacy will be assessed further with the Danish TOFHLA. Additionally, the COPD community nurses will be asked to provide an estimate of the individual level of health literacy among the participants with COPD [52].

Qualitative research interviews will also be conducted with selected participants and/or specialized COPD community nurses during and after trial completion, which will focus on data ethical aspects and user experiences of the telehealth system.

After trial completion, data is extracted comprising data from OpenTeleHealth (physical measurements) and relevant registers (all hospital contacts, all prescribed medicine, all contacts in primary sector, and all social sector contacts) in order to compare the number of exacerbations and the incremental-cost effectiveness ratio from baseline to 6 months follow-up between the intervention- and control group. All data will be stored in REDCap (Research Electronic Data Capture). Only researchers from Aalborg University will have access to the trial dataset.

### Statistical methods

Overall, the statistical methods will follow an intention-to-treat principle [54]. Results from the trial will be presented in accordance with the Consolidated Standards of Reporting Trials (CONSORT) for randomized trial guidelines [55] as well as the Consolidated Health Economic Evaluation Reporting Standards (CHEERS) [56]. Missing data will be subject to multiple imputation [57]. If there is a need for an adjustment of the participants’ baseline characteristics due to differences among the two groups, this will be performed.

To assess the primary outcome, Poisson regression with exponentiated coefficients will be used to produce incidence rate ratios. In assessing HRQoL, generalized linear models [58] will be used to model EQ-5D-5L and the two SF12v2 summary scores; the physical component score (PCS-12) and the mental component score (MCS-12). To assess the secondary outcome of cost-effectiveness, an ICER point estimate will be calculated after 6 months follow-up. The ICER is defined as the difference in the average costs between the two alternatives divided by the difference in average effectiveness between the same alternatives [59]. For the ICER calculation, the effectiveness for both alternatives is assessed based on the utility scores from the EQ-5D-5L questionnaire, with Danish societal weights that are unpublished but under journal peer review [50]. With the help of information on within-trial survival time, QALYs can then be elucidated with linear interpolation of utility scores between baseline and follow-up at six months as the area under this curve [59].

In assessing base-case total costs for both alternatives, this trial adopts a healthcare sector perspective, meaning that the following cost categories are included: additional costs in implementing the algorithm, in- and outpatient hospital costs, medication costs, costs from visiting primary care and general practitioners in particular, community care costs associated with home nurse visits, individual care, and practical help as well as rehabilitation activities in the municipalities. Treatment effects for effectiveness and costs will be calculated separately by applying generalized linear models [58] and will be reported both as an unadjusted estimate and as an estimate that controls for meaningful baseline differences. Finally, the economic evaluation will nuance base-case results by applying both one-way and probabilistic sensitivity analysis (PSA) to allow decision-makers to evaluate the impact of parameter uncertainty that might occur during the trial period on cost-effectiveness at a range of willingness-to-pay levels for an improvement in QALYs [60, 61].

The HLS-EU-Q16 and the Danish TOFHLA provide an individual health literacy score. The HLS-EU-Q16 provides a score ranging from zero (inadequate) to 50 (excellent) and divides the participants into four (HL) groups on this basis. The Danish TOFHLA provides a score ranging from zero (inadequate) to 100 (adequate) and divides the participants into three (HL) groups on this basis. It will be examined whether the division into different groups is similar regardless of health literacy being assessed with the HLS-EU-Q16 or the Danish TOFHLA. One-Way ANOVA will be used to test pre (baseline)-post (follow-up) between-HL-group (as measured with the HLS-EU-Q16) differences in the number of exacerbations. One-Way ANOVA will also be used to test pre (baseline)–post (follow-up) between-HL-group (as measured with the Danish TOFHLA) differences in the number of exacerbations. Furthermore, the specialized COPD community nurses estimate an individual health literacy score among their COPD patients ranging from zero (inadequate) to 10 (adequate) based on their professional judgment. This individual health literacy score provided by the specialized COPD community nurses divides the participants into three (HL) groups. One-Way ANOVA will be used to test pre (baseline)–post (follow-up) between-HL-group differences in the number of exacerbations. It should be noted that the individual health literacy score provided by the specialized COPD community nurses will be compared to the scores obtained with both the HLS-EU-Q16 and the Danish TOFHLA to examine if their professional judgment with regards to health literacy is in line with the standardized scores provided by these tests.

Descriptive statistics will be conducted for the D-TUQ by examining the range, median, and frequency of responses for each item including plots. The questionnaire check marks are listed on a Likert scale ranging from one (disagree) to seven (agree) indicating each participants’ level of agreement with the questions.

### Sample size

The sample size calculation is based on the primary outcome. It is assumed that the control group experience an average of two exacerbations, and the intervention group experienced one exacerbation during the trial period from baseline to the 6-months follow-up. With a power of 90%, a variance of two exacerbations for both groups, a two-sided significance level of 0.05, the required sample size is estimated to be at least 85 participants in each group [62]. Based on experience from the original large-scale trial, TeleCare North [34], a loss to follow-up of 15% in each group is expected. Therefore, the total required sample size in each arm is the estimated to be approximately 100 participants.

## Discussion

In this prospective, parallel, two-armed randomized controlled trial, a COPD prediction algorithm will be tested in a telehealth system with an aim to predict exacerbations in a timely manner. The COPD prediction algorithm is embedded into the general offer of telehealth to patients with COPD in the North Denmark Region. The COPD prediction algorithm will be used to support specialized COPD community nurses in their clinical decisions by alerting them regarding any deviations from the participants’ expected physiological parameters, which may indicate potential exacerbations. The COPD prediction algorithm is expected to improve the telehealth intervention, because the algorithm will use time-series data obtained through weekly repeated average measurements of physiological parameters. In addition, the frequency of the specialized COPD community nurses’ monitoring will be enhanced, as the specialized COPD community nurses not only assess data once a week, but are notified if the COPD prediction algorithm alarm is activated.

An expected strength of the present trial is the pragmatic design with minimal interference into usual practice. To our knowledge, this is the first Danish trial that tests a COPD prediction algorithm into a current operational telehealth system. Another strength is that the trial duration includes a mix of seasons, i.e., autumn and winter, which may increase the generalizability of the results as most exacerbations always occur in winter.

The calculated sample size might be difficult to achieve. A large sample size is desirable to power the trial and to enhance the generalizability of the results. To ensure adequate participant enrollment, the specialized COPD nurses will contact all potential participants in their area. Moreover, the inclusion criteria for the trial are relatively open to ensure a sufficient pool of potential participants. The COPD prediction algorithm performance is highly dependent on the two additional oxygen saturation measurements that the participants have to perform weekly. Thus, a potential risk for the results of the trial is that the participants fail to perform the extra measurements. In addition, the performance of the COPD prediction algorithm highly depends on the specialized COPD community nurses' involvement in assessing measurements, when a COPD prediction algorithm alarm is activated. Therefore, the trial seeks to enhance the involvement of the nurses by repeated training and meetings prior to the trial.

Once the trial has been completed, the results will be published in peer-reviewed journals. The results will also be presented for relevant partners, such as the specialized COPD community nurses and the TeleCare North administration office. The trial has the potential to improve the telehealth intervention in patients with COPD in the form of more intelligent, decision-based monitoring. In the future, it could be relevant to test and implement a similar prediction algorithm among other disease groups, as telehealth becomes more relevant for various diseases and patients with multiple comorbidities.

## Dissemination plans

The results of the trial will be published in international scientific journals. All results are reported in anonymized form. Results will be published regardless of whether or not they are accepted by international journals, and whether results are positive, negative, or inconclusive. Authorship will be granted to those who have contributed to the design, conduct, interpretation, and reporting of the trial.

## Data Availability

All data will be stored in REDCap (Research Electronic Data Capture). Only researchers from Aalborg University will have access to the trial dataset.
